# Systemic Antibiotics in Periodontal Treatment of Diabetic Patients: A Systematic Review

**DOI:** 10.1371/journal.pone.0145262

**Published:** 2015-12-22

**Authors:** Caroline Moura Martins Lobo Santos, Ronaldo Lira-Junior, Ricardo Guimarães Fischer, Ana Paula Pires Santos, Branca Heloisa Oliveira

**Affiliations:** 1 Department of Periodontology, School of Dentistry, Rio de Janeiro State University, Rio de Janeiro, Brazil; 2 Department of Community and Preventive Dentistry, School of Dentistry, Rio de Janeiro State University, Rio de Janeiro, Brazil; University of Sheffield, UNITED KINGDOM

## Abstract

**Aim:**

To evaluate the effects of systemic antibiotics in combination with scaling and root planing (SRP) on periodontal parameters, tooth loss and oral health-related quality of life in diabetes patients.

**Materials and Methods:**

Two independent reviewers screened for controlled clinical trials with at least 6-month follow-up in six electronic databases, registers of clinical trials, meeting abstracts and four major dental journals. After duplicates removal, electronic and hand searches yielded 1,878 records; 18 full-text articles were independently read by two reviewers. To evaluate the additional effect of antibiotic usage, pooled weighted mean differences and 95% confidence intervals were calculated using a fixed effects model.

**Results:**

Five studies met the inclusion criteria, four of which were included in meta-analyses. The meta-analyses showed a significant effect favouring SRP plus antibiotic for reductions in mean probing depth (PD) (-0.22 mm [-0.34, -0.11]) and mean percentage of bleeding on probing (BoP) (4% [-7, -1]). There was no significant effect for clinical attachment level gain and plaque index reduction. No study reported on tooth loss and oral health-related quality of life.

**Conclusion:**

Adjunctive systemic antibiotic use in diabetic patients provides a small additional benefit in terms of reductions in mean PD and mean percentage of BoP.

**Registration:**

PROSPERO: CRD42013006389.

## Introduction

Periodontitis is a biofilm-induced chronic inflammatory disorder of teeth supporting structures [1] that affects over 740 million people worldwide in its severe form [2]. Diabetes mellitus is a group of chronic metabolic disorders characterized by irregular glucose metabolism caused by defects in insulin production and/or action [3]. The International Diabetes Federation estimates that approximately 382 million people have diabetes and a 55% increase in its prevalence is expected by the year 2035 [4].

Several clinical studies have established the relationship between diabetes and periodontitis. This relationship appears to be bidirectional, with diabetes being a risk factor for periodontitis [5] and the severity of periodontitis a factor influencing glycemic control and the development of complications in diabetic patients [6]. In addition, periodontal treatment may have a positive effect on glycemic control in diabetic patients [7, 8].

The clinical benefit of nonsurgical periodontal treatment is well documented in terms of probing depth reduction, bleeding on probing reduction and clinical attachment gain [9]. Nevertheless, whether to use systemic antibiotics as an adjunct therapy for periodontal disease in any patient is still a subject of debate. While there is evidence that the use of antibiotics with nonsurgical periodontal therapy provides some benefit to systemically healthy patients [10–12], their use is generally recommended only in specific clinical situations [12]. Also, there is no evidence that there is any additional benefit for smokers with chronic periodontitis [13].

The use of adjunct antibiotics with scaling and root planing in diabetic patients is also a contentious issue [14–16]. The results of individual clinical trials are conflicting or inconclusive [17–19] and an appropriate summary of their results using a systematic approach would clarify whether the use of antibiotics in periodontal treatment could be beneficial to diabetic patients. The aim of this study was to assess the effects of the adjunctive use of systemic antibiotics in nonsurgical periodontal treatment, compared to nonsurgical periodontal treatment alone, on clinical periodontal parameters, tooth loss and oral health-related quality of life in patients with diabetes.

## Materials and Methods

### Study design

A systematic review of controlled clinical trials with a follow-up of at least six months.

### Participants

Type 1 or type 2 diabetes patients, with a clinical diagnosis of periodontitis, regardless of classification. Chronic and aggressive periodontitis were considered for inclusion with no age restriction. Gestational diabetes was not considered for inclusion.

### Interventions

Scaling and root planing (SRP) combined with adjunctive use of systemic antibiotics or sub-antimicrobial doxycycline (SDD) versus SRP alone, with or without a placebo. Quadrant-wise and full-mouth periodontal treatments were considered for inclusion. Any type of systemic antibiotic or combinations of antibiotics, as well as different therapeutic regimens were eligible for inclusion. Studies were excluded when systemic antibiotics were used only in periodontal supportive therapy or with co-interventions such as slow-release devices or subgingival irrigation with antimicrobials.

### Outcomes

Changes in periodontal parameters (i.e., clinical attachment level, probing depth, plaque index, and bleeding on probing), tooth loss, and oral health-related quality of life. The occurrence of side effects associated with antibiotics was also investigated.

### Search strategy

The following databases were searched from their earliest records through May 2015: The Cochrane Central Register of Controlled Trials (CENTRAL), MEDLINE via PubMed, EMBASE, LILACS, BBO (Brazilian Dental Library), and the Brazilian database of theses and dissertations (*Banco de Teses CAPES*). The strategy was developed for MEDLINE using controlled vocabulary, with words derived from "Medical Subject Headings" (MeSH) associated with free terms considered relevant to the topic in question; the asterisk symbol (*) was used for truncation (S1 Appendix). This strategy was adapted for the other databases without any idiom restrictions.

The following international registers of ongoing trials were also evaluated: ClinicalTrials.gov, Current Controlled Trials, *Registro Brasileiro de Ensaios Clínicos* (ReBEC), EU Clinical Trials Register, and Australian New Zealand Clinical Trials Registry. Hand searches were performed in the Journal of Clinical Periodontology (since 2003), Journal of Periodontology (since 2003), Journal of Periodontal Research (since 2004), and Journal of Dental Research (since 2004). Searches in journals included in the Cochrane Master List of Journals Being Searched were examined from the last update onward. Likewise, abstracts from the International Association for Dental Research (2001–2014) and the European Federation of Periodontology meetings (EuroPerio 4, 5, 6 and 7) were examined.

### Data collection and analysis

Two independent reviewers (CMMLS and RLJ) read the titles and abstracts, when available, of all records identified. Whenever a study seemed to meet the inclusion criteria, but complete information was lacking, the full-text article was obtained. The same reviewers independently extracted the data on characteristics of the study population, interventions, outcomes, and length of follow-up using a data extraction form. Attempts were made to contact authors to check on incomplete data. Disagreements were solved by consulting two other reviewers (BHO and APPS). Studies meeting the inclusion criteria were obtained in full.

We used the Cochrane Collaboration tool for assessing risk of bias in the studies included [20]. The following domains were evaluated as having low, high or unclear risk of bias: sequence generation, allocation concealment, blinding, incomplete outcome data, selective outcome reporting and other biases, such as losses to follow-up (low risk of bias when losses were non-differential and less than 20%), diagnosis reliability (low risk of bias when good) [21], and comparability between groups at baseline (low risk of bias if groups were balanced in terms of age, gender, level of metabolic control and severity of periodontitis).

Meta-analyses of the differences in six-month means were performed. Heterogeneity of studies was assessed by visual inspection of forest plots, Chi-square (χ^2^) test for heterogeneity and Higgins index (I^2^). A fixed-effects model was used in the absence of heterogeneity (χ^2^ with significance level >0.10 and I^2^ <50%). Post-hoc sensitivity analyses were performed excluding studies restricted to non-smokers and studies restricted to patients with good metabolic control. All analyses were carried out in Stata® 11 (StataCorp LP, College Station, Tex., USA).

## Results

**Fig 1:**
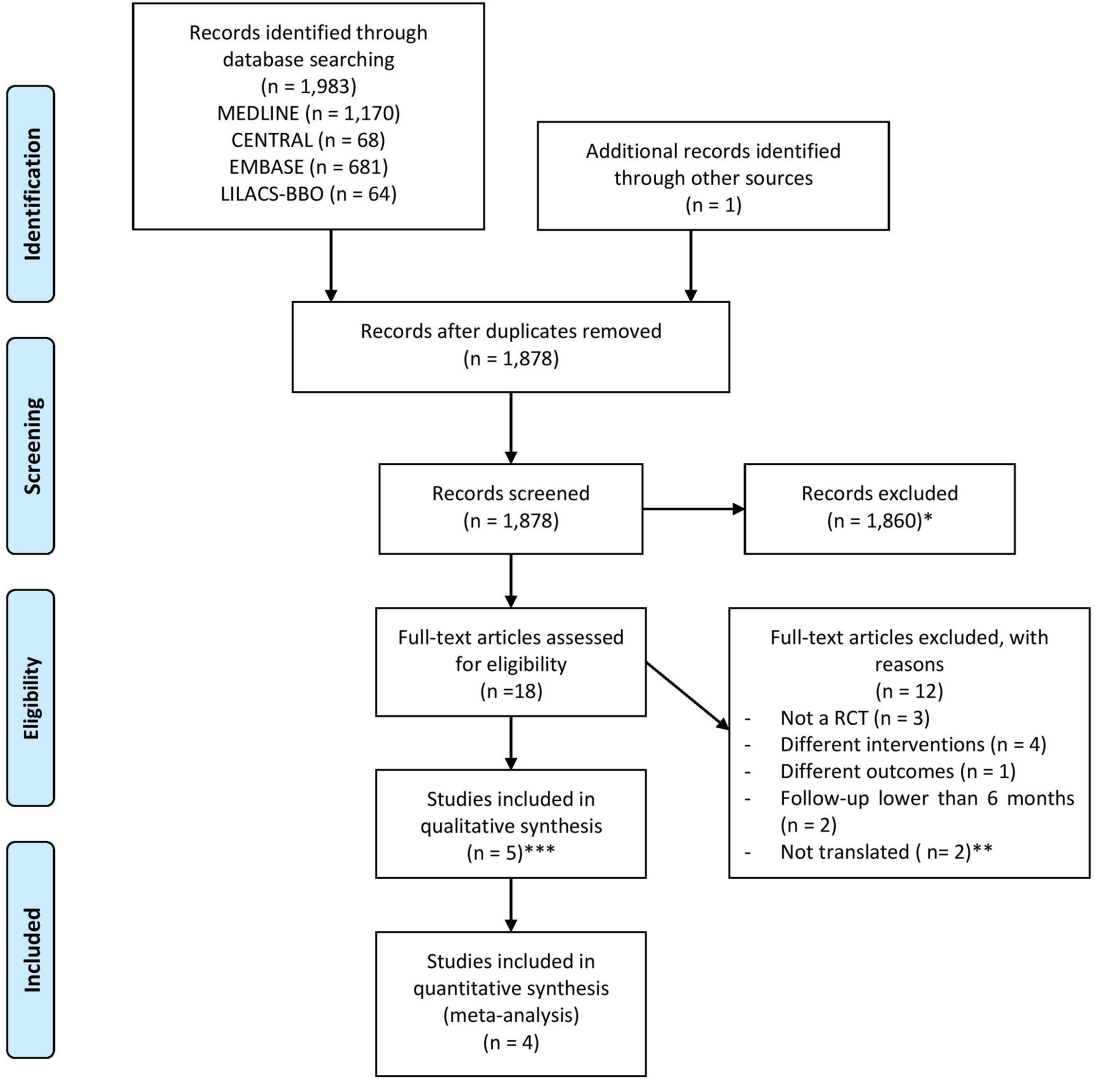
Flow diagram showing the process of identifying, screening, assessing for eligibility, excluding and including the studies retrieved from the electronic and hand searches. *It was not possible to obtain one full-text article and 1,859 did not meet the inclusion criteria. **One Chinese and one Russian. ***Two articles using the same population were merged and only one article was included.

The electronic search retrieved 1,983 records. One additional record was found after hand searching an IADR meeting abstract. After excluding duplicates, there were 1,878 records, 1,859 of which did not meet the inclusion criteria, and 19 were selected for full reading, but one full-text could not be obtained. Thus, 18 full-text were read and assessed for eligibility. Five studies met the inclusion criteria and four were used in meta-analyses. Fig 1 describes the studies identified, screened, assessed for eligibility, excluded and included in the review. The list of 12 excluded studies and the reasons for their exclusion are presented in S2 Appendix.

The general characteristics of the studies are presented in Table 1. The studies used different antibiotics and/or therapeutic regimens. The Deo et al. (2010) [22] trial used SDD and therefore was not included in the meta-analysis. In that study, a significantly greater PD reduction and CAL gain were found for the group taking SDD (3.06 mm versus 2.54 mm for PD and 2.25 mm versus 1.58 mm for CAL). Deo et al. (2010) [22] described no adverse reactions to SDD therapy.

**Table 1:** General characteristics of included studies.

Study (Country)	Design	Population	Number of included patients	Interventions	Compliance assessment	Study duration (follow-up points)
Grossi et al. 1996 (USA)	RCT	42 essentially type 2 poorly controlled diabetic with severe periodontitis	Test = 18	Test = SRP + H_2_0 irrigation + systemic doxycycline 100 mg/day for 2 weeks	Not reported	12 months (3, 6, and 12 months)
			Control = 24	Control = SRP + H_2_0 irrigation + placebo		
Deo et al. 2010 (India)	RCT	20 diabetic patients with chronic periodontitis (mean age: 37.1 ± 3.96)	Test = 10	Test = SRP + doxycycline hyclate 20 mg twice a day for the treatment period of six months	Not reported	6 months
			Control = 10	Control = SRP + placebo capsule b.i.d. for the treatment period of six months		
Botero et al. 2013 (Colombia)	Double blind RCT	70 type 1 and type 2 poorly controlled diabetics patients with moderate periodontitis (mean age: test = 55.9±12.6; control = 58.2±11.1)	Test = 33	Test = SRP + azithromycin 500mg/day for 3 days	Not reported	9 months (3, 6, and 9 months)
			Control = 37	Control = SRP + placebo 500mg/day for 3 days		
Miranda et al. 2014 (Brazil)	Double blind RCT	56 type 2 poorly controlled diabetics with generalized chronic periodontitis (mean age: test = 54.0±8.2; control = 53.7±8.0)	Test = 29	Test = SRP + MTZ (400 mg thrice a day [TID] for 14 days) + AMX (500 mg TID for 14 days)	3 control subjects and one test subject had 1 or 2 pills left in the bottles on Day 14	12 months (3, 6, and 12 months)
			Control = 27	Control = SRP + placebo for 14 days		
Tsalikis et al. 2014 (Greece)	Double blind RCT	66 type 2 well controlled diabetics with moderate or advanced periodontitis (mean age: test = 62.9±10.0; control = 57.9±8.2)	Test = 31	Test = SRP + doxycycline (200 mg as loading dose and 100 mg for 20 days)	All patients followed the prescribed regimen	6 months (3 and 6 months)
			Control = 35	Control = SRP + placebo with the same instructions		

RCT = randomized clinical trial. SRP = scaling and root planing. MTZ = metronidazole. AMX = amoxicillin.

**Fig 2:**
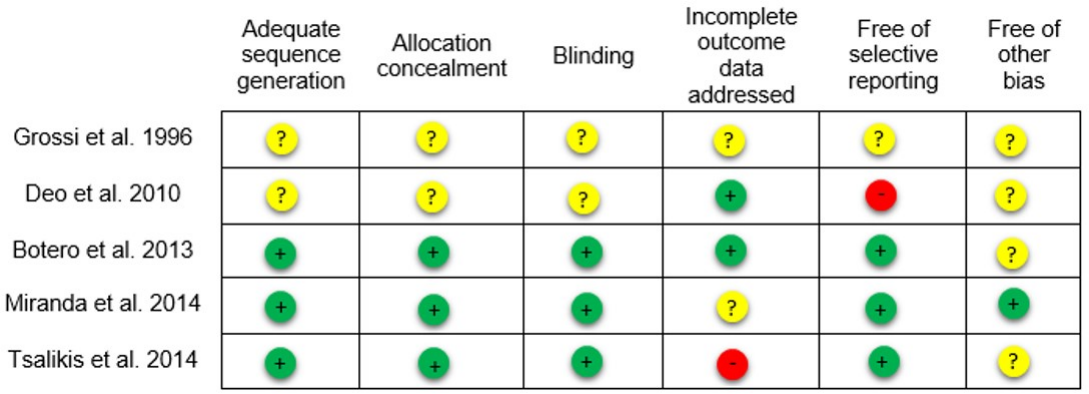
Ascertainment of the risk of bias in the included studies. + = yes;— = no;? = uncertain.

All studies presented data on probing depth, clinical attachment level and bleeding on probing. Tsalikis et al. (2014) [19] did not present data on plaque index. For the study by Grossi et al. (1996) [23], additional information on sample size and outcomes were obtained by e-mail. Two studies assessed only type 2 diabetic patients [18, 19]. Follow-up varied from 6 to 12 months. No study evaluated tooth loss and oral health-related quality of life. Fig 2 shows the risk of bias in the included studies. No study had low risk of bias in all domains evaluated.

**Fig 3:**
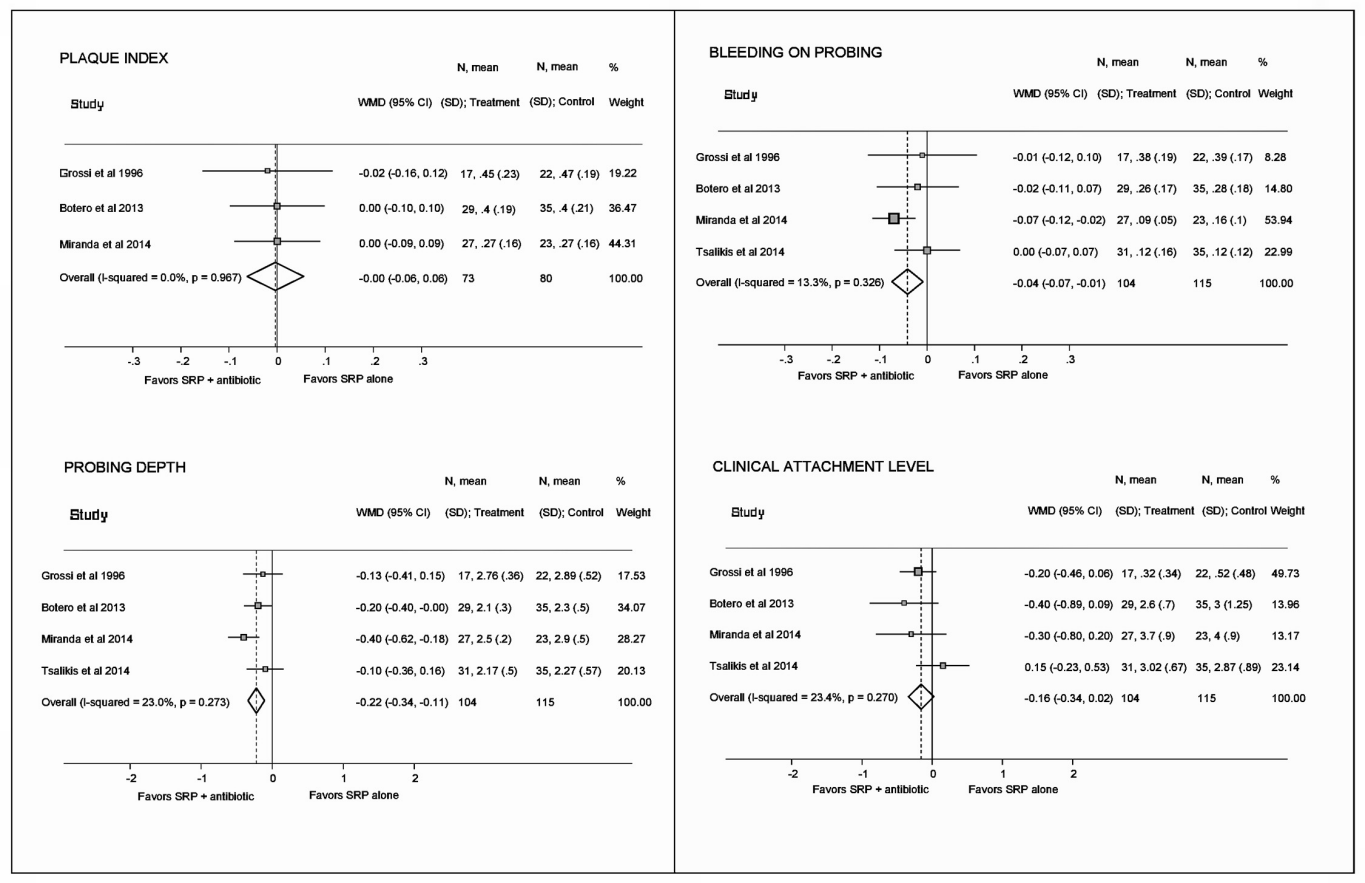
Forest plots presenting pooled weighted mean differences and 95% confidence intervals for periodontal parameters between test and control groups.

Meta-analyses showed a significant effect favoring SRP plus antibiotic for reductions in mean PD (-0.22 mm [-0.34, -0.11]) and mean percentage of BoP (-4% [-7, -1]). There was no significant effect for CAL gain and PI reduction (Fig 3). Post-hoc sensitivity analyses showed a significant gain in CAL when studies restricted to non-smokers (-0.24 mm [-0.47, -0.02]) or restricted to well-controlled patients (-0.25 mm [-0.46, -0.05]) were excluded; both results favored SRP plus antibiotic (S3 Appendix).

Three studies reported on side effects related to the use of antibiotics or placebo [17–19]. In the Botero et al. (2013) [17] trial, only one control patient reported gastrointestinal discomfort. Miranda et al. (2014) [18] found more side effects in the test group (diarrhea = 7; headache = 4; metallic taste = 4; and nausea/vomiting = 5) than in the control group (diarrhea = 3; headache = 1; metallic taste = 2; and nausea/vomiting = 2). One control patient reported dizziness and difficulty swallowing in the Tsalikis et al. (2014) [19] study.

## Discussion

Recent systematic reviews have shown that the use of adjunctive antibiotics may improve periodontal healing in systemically healthy patients with periodontitis [10, 12, 24], although the clinical relevance of this improvement is still subject to debate. To the best of our knowledge, this is the first systematic review of the additional clinical benefit of adjunctive use of systemic antibiotics over SRP alone in diabetic patients. Our results showed an additional 0.22 mm reduction in mean PD and a 4% reduction in mean BoP for SRP plus antibiotic. No additional benefits were observed for mean CAL gain and mean PI reduction.

These findings should be interpreted with great caution. Despite the statistical significance, the clinical relevance of six-month reductions of 0.22 mm in mean PD and of 4% in mean BoP is doubtful. In addition, there is a concern about the increasing use of antibiotics worldwide. From 2000 to 2010, global use of antibiotics increased by 36% [25], contributing to the spread of antimicrobial resistance. Antibiotics prescribed by dentists affect microorganisms beyond the oral cavity [26] and the greater the number or duration of antibiotic courses, the greater the rates of resistance [27]. The prescription of antibiotics by dentists should be balanced against the risks of increasing antimicrobial resistance. Thus, the lack of evidence of a clinically relevant benefit argues against such use in the periodontal treatment of diabetic patients.

Averaging whole mouth PD and CAL may underestimate the benefits of adjunctive antibiotics. The positive results are more pronounced when analyzing deep pockets [12]. Miranda et al. (2014) [18] found increased reduction in numbers of sites with PD ≥ 6 mm in the group taking amoxicillin/metronidazole, which seems to be effective in combination with scaling and root planing in the treatment of chronic periodontitis [10]. Further studies are needed to ascertain the benefit of adjunctive antibiotics based on disease severity.

Our meta-analyses were based on six-month results. In order to be considered a useful intervention, the clinical benefit of antibiotics should remain steady over time. Botero et al. (2013) [17] and Miranda et al. (2014) [18] found stable results 9 and 12 months, respectively, after SRP plus systemic antibiotic. The ideal frequency of antibiotic courses and the extent of the possible long-term benefits of this therapeutic approach remain to be determined.

The decision to use systemic antibiotics should take into consideration, in addition to the other points discussed, whether the clinical benefits outweigh the potential side effects. No study reported serious adverse events related to the use of antibiotics. One study performed a comparative analysis between test and control groups. A higher frequency of side effects was found in the antibiotic group, but the difference was not statistically significant [18].

The risk of bias in the studies included is also worth noting. Among the trials included in the meta-analyses, one had high risk of bias [19] and three were at unclear risk of bias [17, 18, 23]. Deo et al. (2010) [22] had high risk of bias.

Only one study [22] used SDD, which does not have anti-microbial effects and is used as host-modulator agent [28], hence it was not included in our meta-analysis. Although this study found positive results favoring SDD therapy for PD and CAL, no definite conclusion can be drawn due to the small number of participants and unclear risk of bias.

One limitation of our meta-analyses was the inclusion of different types and regimens of antibiotics. Recently, Keestra et al. (2015) [12] reported that no type of antibiotic was superior to any other. However, when analyzing moderate and deep pockets, they found a trend suggesting that amoxicillin/metronidazole is more effective. Our PD and BoP results were largely influenced by Miranda et al. (2014) [18], the only study using the amoxicillin/metronidazole combination.

Another limitation was the diversity in tobacco exposure and the level of metabolic control across the studies. It is well known that smoking has a negative influence on the outcome of periodontal therapy [29] and level of metabolic control is an important mediator of the relationship between diabetes and periodontitis [30]. Although baseline severity of periodontal disease was similar in all studies, we performed sensitivity analyses excluding studies restricted to non-smokers [18, 19] and studies restricted to patients with good metabolic control [19]. When studies restricted to non-smokers were excluded, statistical significance remained unchanged for PI and PD. However, the confidence interval for BoP became wider, suggesting no evidence that the adjunctive use of antibiotics provides any benefit. On the other hand, the pooled estimate for CAL became statistically significant, favoring the adjunctive use of antibiotics (S3 Appendix). When the only study that was restricted to patients with good metabolic control was excluded, results for BoP and PD did not change. Again, the pooled estimate for CAL became statistically significant, favoring the adjunctive use of antibiotics (S3 Appendix). These uncertainties around the effect estimates reinforce the need for better evidence on this subject in order to inform clinical practice.

Despite differences in types and regimens of antibiotics, inclusion of smokers, type of diabetes and levels of metabolic control, no evidence of statistical heterogeneity was found. Due to the small number of studies included, it was not possible to perform meta-regression to evaluate the potential modifier effect of covariates. One study has included topical irrigation with water in both groups [23]. Although we have excluded studies using co-interventions, water irrigation was accepted since no additional benefit in PD reduction and CAL gain has been shown due to its use in diabetic patients [31].

Unfortunately, we did not find any trial that provided data on true outcome measures, such as tooth loss, adverse events, oral health-related quality of life and bacterial resistance. Cunha-Cruz et al. (2008) [32], in a large retrospective cohort study, found no association between systemic antibiotics prescribed for dental or medical reasons and lower incidence of tooth loss. Well-designed clinical trials with large sample sizes, longer follow-up periods and true outcome measures need to be given high priority. Also, as compliance with antibiotic regimen is important to treatment outcomes, all studies should report data on it.

This study indicates that adjunctive systemic antibiotic use provides statistically significant benefits in terms of reductions in mean probing depth and mean percentage of bleeding on probing, but no improvement in CAL gain. However, the widespread use of systemic antibiotics in periodontal treatment of diabetics should not be encouraged, since the clinical relevance of these findings has yet to be shown. The risk of bias of included studies should also be taken into account when translating the results into clinical practice.

Based on the results of this systematic review, we consider that it is still appropriate to adopt a conservative approach in the prescription of systemic antibiotics for the treatment of chronic periodontitis in diabetic patients, in line with what is currently recommended to other chronic periodontal patients [33].

## Supporting Information

S1 Appendix Search strategy for MEDLINE via PubMed. (PDF)

S2 Appendix List of excluded studies and the reasons for exclusion. (PDF)

S3 Appendix Sensitivity analyses. (PDF)

S4 Appendix Prisma checklist. (PDF)
