# Supplementary material for: Systemic Antibiotics in Periodontal Treatment of Diabetic Patients: A Systematic Review
Source: PLoS One. 2015 Dec 22;10(12):e0145262. doi: 10.1371/journal.pone.0145262 (PMC4687852; doi:10.1371/journal.pone.0145262)
Supplement: S1 Appendix — (PDF) [file pone.0145262.s001.pdf]

S1 Appendix. Search strategy for MEDLINE via PubMed.

#1 periodontal disease* (MeSH term); or periodontal index* (MeSH term); or periodont* (All fields); or attachment loss (All fields); or bone loss* (All fields)

#2 dental prophylaxis (MeSH term); or subgingival curettage* (MeSH term); or periodontal treatment (All fields); or periodontal therapy (All fields); or (scaling and root planing) (All fields); or nonsurgical periodontal treatment (All fields); or non-surgical periodontal treatment (All fields); or non surgical periodontal treatment (All fields); or full-mouth debridement (All fields); or tooth scaling (All fields); or teeth scaling (All fields); or subgingival scaling (All fields)

#3 diabetes mellitus (MeSH term); or hemoglobin a, glycosylated (MeSH term); or hyperglycemia* (MeSH term); or glucose (MeSH term); or insulin resistance (MeSH term); or diabet*(All fields); or mellitus (All fields); or diabetic patient (All fields); or type 1 diabetes (All fields); or type 2 diabetes (All fields); or DM (All fields); or DM1 (All fields); or DM2 (All fields)

#1 and #2 and #
