# Supplementary material for: Systemic Antibiotics in Periodontal Treatment of Diabetic Patients: A Systematic Review
Source: PLoS One. 2015 Dec 22;10(12):e0145262. doi: 10.1371/journal.pone.0145262 (PMC4687852; doi:10.1371/journal.pone.0145262)
Supplement: S2 Appendix — (PDF) [file pone.0145262.s002.pdf]

S2 Appendix. List of excluded studies and the reasons for exclusion.

Reference	Reason for exclusion
Albrecht M, Bánóczy J, Gyenes V, Ember G, Rigó O, Valkovics M, Tamas G. [Treatment of gingivitis and periodontal diseases with insadol in diabetics]. Fogorvosi Szemble. 1988 Mar;81(3):65-71. [Article in Hungarian]	Different interventions
Bay I, Ainamo J, Gad T. The response of young diabetics to periodontal treatment. J Periodontol. 1974 Nov;45(11):806-8.	Different interventions
Chee HK, Lim LP, Tay F, Thai AC, Sum CF. Non-surgical periodontal therapy and serum lipid levels in patients with diabetes mellitus. Ann R Australas Coll Dent Surg. 2006 Sep;18:46.	Different interventions
Chee HK, Lim LP, Tay F, Thai AC, Sum CF. Non-surgical periodontal treatment and lipid levels in diabetic patients. Ann R Australas Coll Dent Surg. 2008 Jun;19:183.	Different interventions
Fett KD, Jutzi E. [DENTITION OF DIABETES. EFFECT OF THE TREATMENT METHODS ON THE NUMBER OF TEETH. II]. Dtsch Zahnarztl Z. 1965 Aug 1;20:902-6. [Article in German]	Not a RCT
Fett KD, Jutzi E. [Periodontopathies in diabetics--brief contribution to the etiology and therapy with Parodontal-F5]. Dtsch Stomatol. 1965 Oct;15(10):730. [Article in German]	Not a RCT
Fursa VT. [Treatment of periodontal diseases in diabetes mellitus]. Med Sestra. 1982 Mar;41(3):14-7. [Article in Russian]	Not translated
Fursa VT, Shelepov AS. [Effect of treatment on certain protein and carbohydrate components of blood serum and saliva in patients with diabetes mellitus complicated by periodontosis]. Vrach Delo. 1975 Jan;(1):35-7. [Article in Russian]	Different outcomes
Han B, You L. [Effect of initial periodontal therapy on diabetic patients with chronic periodontitis]. Zhonghua Kou Qiang Yi Xue Za Zhi. 2010 May;45(5):282-6. [Article in Chinese]	Not translated
Llambés F, Silvestre F-J, Hernández-Mijares A, Guiha R, Caffesse R. Effect of nonsurgical periodontal treatment with or without doxycycline on the periodontium of type 1 diabetic patients. J Clin Peridontol 2005; 32: 915–920.	Follow-up lower than 6-months
Llambés F, Silvestre FJ, Hernández-Mijares A, Guiha R, Bautista D, Caffesse R. Efect of periodontal disease and non surgical periodontal treatment on C-reactive protein. Evaluation of type 1 diabetic patients	Follow-up lower than 6-months
Vaĭnshteĭn NV, Zinger MA, Parovchenko AA. [Comparison of different types of sugar curves with the severity of parodontosis and the effectiveness of treatment]. Stomatologiia (Mosk). 1969 Jul-Aug;48(4):21-4. [Article in Russian]	Not a RCT
