# Supplementary material for: Systemic Antibiotics in Periodontal Treatment of Diabetic Patients: A Systematic Review
Source: PLoS One. 2015 Dec 22;10(12):e0145262. doi: 10.1371/journal.pone.0145262 (PMC4687852; doi:10.1371/journal.pone.0145262)
Supplement: S3 Appendix — (PDF) [file pone.0145262.s003.pdf]

S3 Appendix. Sensitivity analyses.

Analyses after exclusion of the studies restricted to non-smokers.

- *Plaque index (PI)*

Study	WMD	[95% Conf. Interval]		% Weight
Grossi et al 1996	-0.020	-0.155	0.115	34.51
Botero et al 2013	0.000	-0.098	0.098	65.49
I-V pooled WMD	-0.007	-0.086	0.072	100.00

Heterogeneity chi-squared = 0.06 (d.f. = 1) p = 0.814
I-squared (variation in WMD attributable to heterogeneity) = 0.0%
Test of WMD=0 : z= 0.17 p = 0.865

- *Bleeding on probing (BoP)*

Study	WMD	[95% Conf. Interval]		% Weight
Grossi et al 1996	0.000	-0.111	0.111	37.39
Botero et al 2013	-0.020	-0.106	0.066	62.61
I-V pooled WMD	-0.013	-0.081	0.055	100.00

Heterogeneity chi-squared = 0.08 (d.f. = 1) p = 0.780
I-squared (variation in WMD attributable to heterogeneity) = 0.0%
Test of WMD=0 : z= 0.36 p = 0.718

- *Probing depth (PD)*

Study	WMD	[95% Conf. Interval]		% Weight
Grossi et al 1996	-0.130	-0.407	0.147	33.97
Botero et al 2013	-0.200	-0.398	-0.002	66.03
I-V pooled WMD	-0.176	-0.337	-0.015	100.00

Heterogeneity chi-squared = 0.16 (d.f. = 1) p = 0.687
I-squared (variation in WMD attributable to heterogeneity) = 0.0%
Test of WMD=0 : z= 2.14 p = 0.032

- *Clinical attachment level (CAL)*

Study	WMD	[95% Conf. Interval]		% Weight
Grossi et al 1996	-0.200	-0.458	0.058	78.08
Botero et al 2013	-0.400	-0.886	0.086	21.92
I-V pooled WMD	-0.244	-0.471	-0.016	100.00

Heterogeneity chi-squared = 0.51 (d.f. = 1) p = 0.476
I-squared (variation in WMD attributable to heterogeneity) = 0.0%
Test of WMD=0 : z= 2.10 p = 0.036

Analyses after exclusion of the study restricted to patients with good metabolic control.

- *Bleeding on probing (BoP)*

Study	WMD	[95% Conf. Interval]		% Weight
Grossi et al 1996	0.000	-0.111	0.111	11.39
Botero et al 2013	-0.020	-0.106	0.066	19.08
Miranda et al 2014	-0.070	-0.115	-0.025	69.53
I-V pooled WMD	-0.052	-0.090	-0.015	100.00

Heterogeneity chi-squared = 0.51 (d.f. = 1) p = 0.476
I-squared (variation in WMD attributable to heterogeneity) = 0.0%
Test of WMD=0 : z= 2.10 p = 0.036

- *Probing depth (PD)*

Study	WMD	[95% Conf. Interval]		% Weight
Grossi et 1996	-0.130	-0.407	0.147	21.95
Botero et al 2013	-0.200	-0.398	-0.002	42.66
Miranda et al 2014	-0.400	-0.618	-0.182	35.39
I-V pooled WMD	-0.255	-0.385	-0.126	100.00

Heterogeneity chi-squared = 2.78 (d.f. = 2) p = 0.249
I-squared (variation in WMD attributable to heterogeneity) = 28.1%
Test of WMD=0 : z= 3.86 p = 0.000

- *Clinical attachment level (CAL)*

Study	WMD	[95% Conf. Interval]		% Weight
Grossi et al 1996	-0.200	-0.458	0.058	64.70
Botero et al 2013	-0.400	-0.886	0.086	18.16
Miranda et al 2014	-0.300	-0.801	0.201	17.14
I-V pooled WMD	-0.253	-0.461	-0.046	100.00

Heterogeneity chi-squared = 0.55 (d.f. = 2) p = 0.760
I-squared (variation in WMD attributable to heterogeneity) = 0.0%
Test of WMD=0 : z= 2.40 p = 0.017
